# Supplementary figures and images for: Clock genes regulate mating activity rhythms in the vector mosquitoes, Aedes albopictus and Culex quinquefasciatus
Source: PLoS Negl Trop Dis. 2022 Dec 1;16(12):e0010965. doi: 10.1371/journal.pntd.0010965 (PMC9746994; doi:10.1371/journal.pntd.0010965)

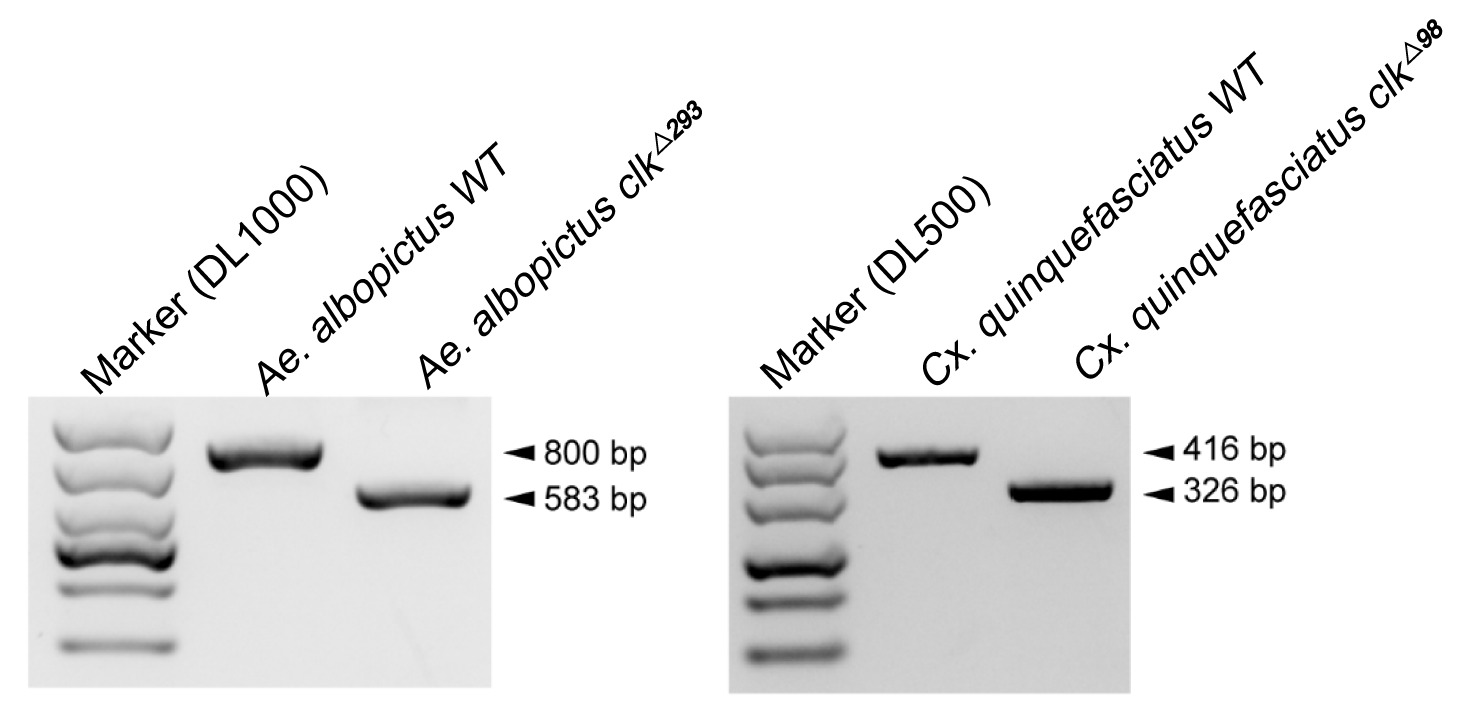

Supplement: S1 Fig — Genomic DNA extracted from Ae. albopictus clkΔ293 and Cx. quinquefasciatus clkΔ98 and mutations were confirmed with PCR. (TIF) [file pntd.0010965.s001.tif]

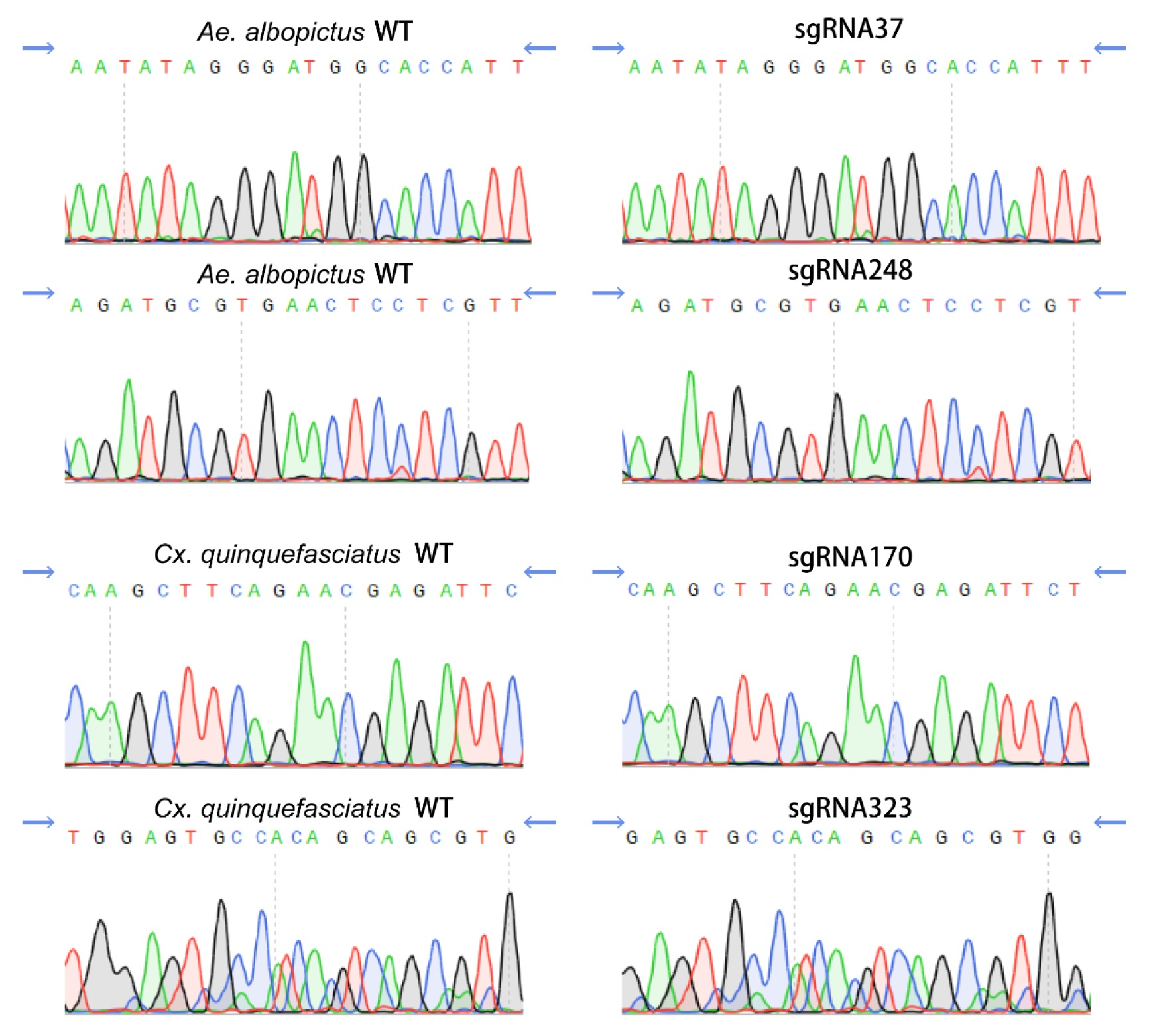

Supplement: S2 Fig — Potential off-target mutations were screened in genomic DNA extracted from Ae. albopictus clkΔ293 and Cx. quinquefasciatus clkΔ98 mutants. Blue arrows indicate the off-target test primers. No off-target mutations were confirmed. (TIF) [file pntd.0010965.s002.tif]

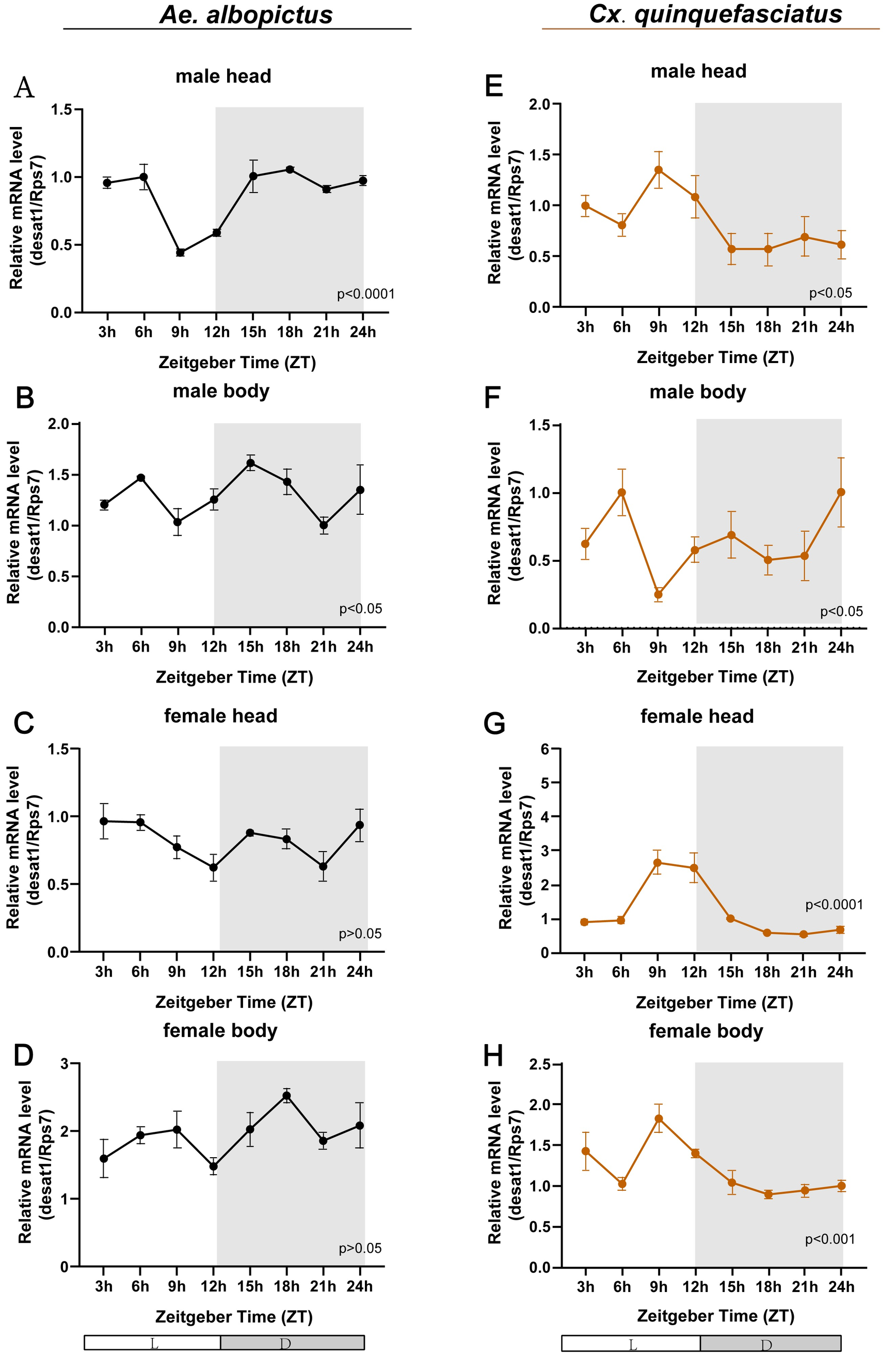

Supplement: S3 Fig — (A) Ae. albopictus male heads, (B) Ae. albopictus male bodies, (C) Ae. albopictus female heads, (D) Ae. albopictus female bodies, (E) Cx. quinquefasciatus male heads, (F) Cx. quinquefasciatus male bodies, (G) Cx. quinquefasciatus female heads, (H) Cx. quinquefasciatus female bodies. Tissues were collected from 3 replicate groups with 10 individuals at each zeitgeber time with 3h intervals for 24 h under LD condition. RNA levels were quantified by qPCR. Each value was the mean±SEM. White and black shades represent the photophase and scotophase, respectively. P-value determined by one-way ANOVA. (TIF) [file pntd.0010965.s003.tif]

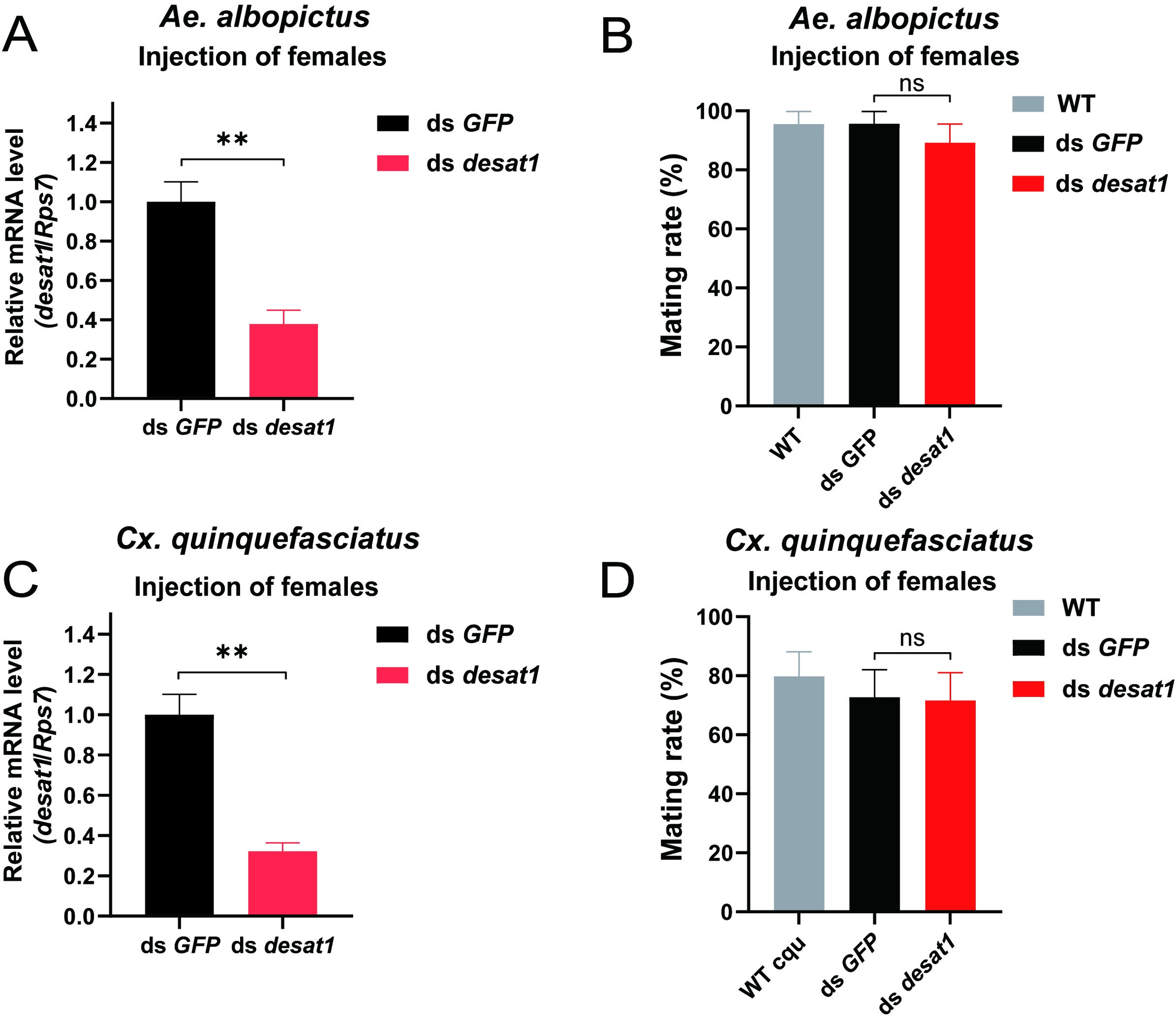

Supplement: S4 Fig — (A) Silencing efficiency of desat1 of female Ae. albopictus on 4dpi. (B) Mating rate of Ae. albopictus. (C) Silencing efficiency of desat1 of female Cx. quinquefasciatus on 4dpi. (D) Mating rate of Cx. quinquefasciatus. A total of 30 injected females on 4 dpi were exposed to 30 virgin males at ZT9 and they were exposed for 24h. Statistics were performed using GLM with binomial distribution and error bars represent 95% confidence intervals (CIs). Each mosquito was measured only once. n = 88–90 for each group, ** P < 0.01, ns = not significant. (TIF) [file pntd.0010965.s004.tif]

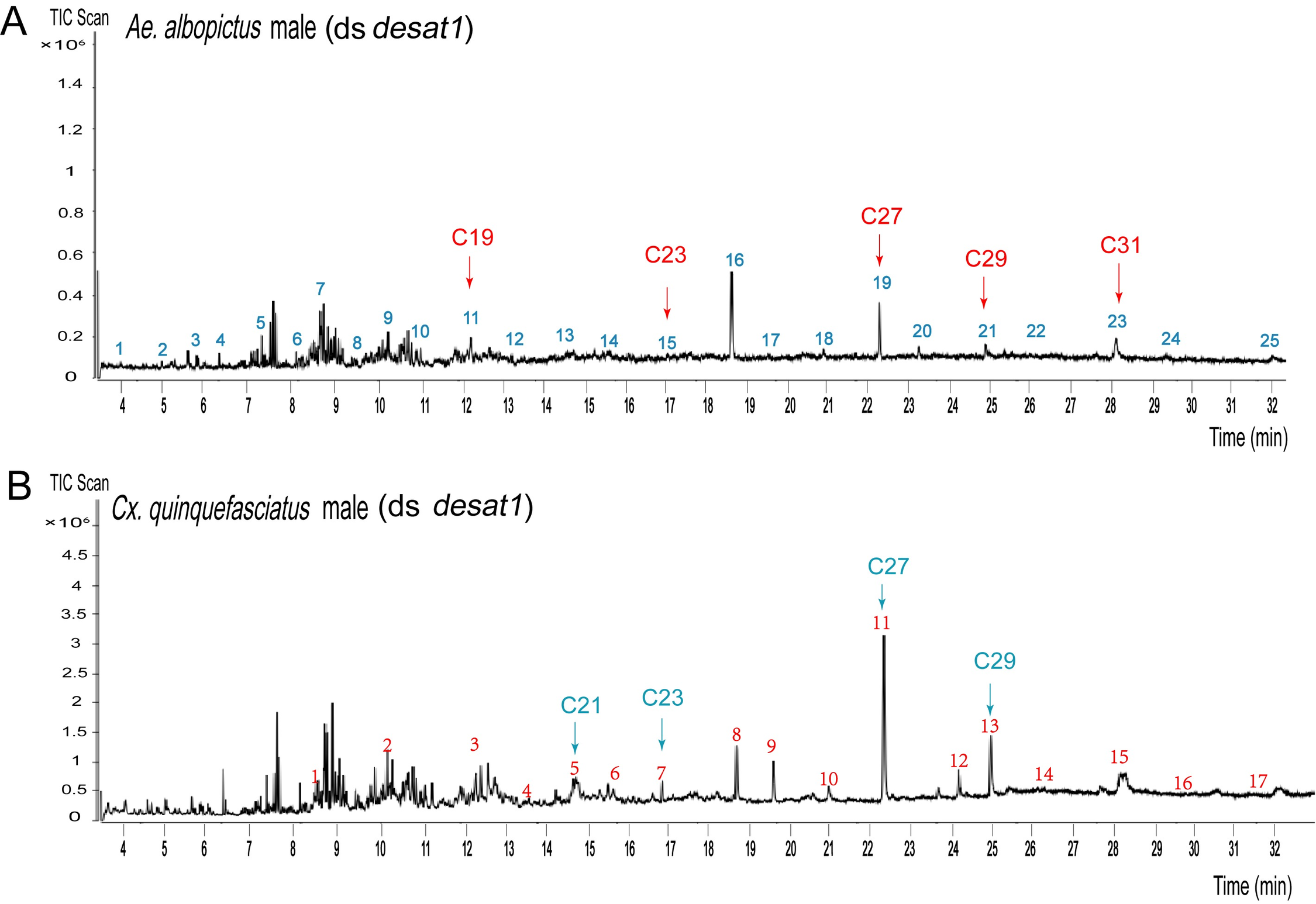

Supplement: S5 Fig — (A-B) Representative CHC profiles of male Ae. albopictus (A) and Cx. quinquefasciatus (B) on day 4 after desat1 ds RNA injection. Numbers above the peaks correspond to peak numbers given in S3 Table and S4 Table. (TIF) [file pntd.0010965.s005.tif]

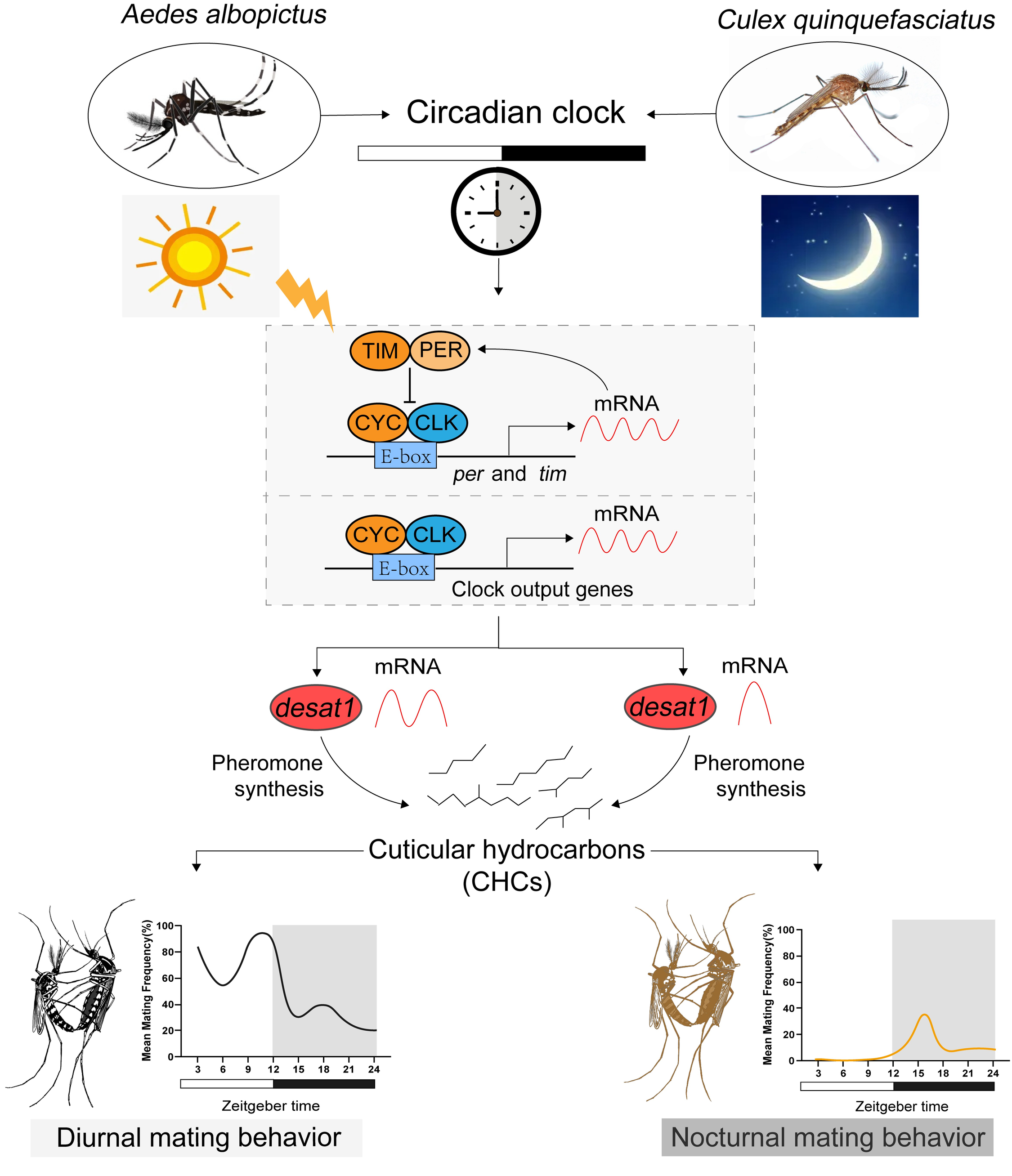

Supplement: S6 Fig — (TIF) [file pntd.0010965.s006.tif]
