# Supplementary material for: Clock genes regulate mating activity rhythms in the vector mosquitoes, Aedes albopictus and Culex quinquefasciatus
Source: PLoS Negl Trop Dis. 2022 Dec 1;16(12):e0010965. doi: 10.1371/journal.pntd.0010965 (PMC9746994; doi:10.1371/journal.pntd.0010965)
Supplement: S1 Table — (DOCX) [file pntd.0010965.s007.docx]

**S1 Table. Statistics of mutation rates of G_0_ adults and G_1_ mutant pools in *Ae. albopictus* and *Cx. quinquefasciatus***

| Group | Embryos injected | Hatched (%) | Pupated (%) | G_0_ adult survivors (%) | G_0_ mosaic (%) | No. of pools | G_1_ mutant pools |
| --- | --- | --- | --- | --- | --- | --- | --- |
| *Ae. albopictus Clk* | 294 | 25.17%  (74/294) | 90.54%  (67/74) | 88.06%  (59/67) | 7.78%  (4/59) | 4 | 50.00%  (2/4) |
| *Cx. quinquefasciatus Clk* | 938 | 20.90%  (196/938) | 96.43% (189/196) | 83.60% (158/189) | 12.03%  (19/158) | 19 | 5.26%  (1/19) |
