# Supplementary material for: Clock genes regulate mating activity rhythms in the vector mosquitoes, Aedes albopictus and Culex quinquefasciatus
Source: PLoS Negl Trop Dis. 2022 Dec 1;16(12):e0010965. doi: 10.1371/journal.pntd.0010965 (PMC9746994; doi:10.1371/journal.pntd.0010965)
Supplement: S2 Table — Mismatches bases are highlighted in lowercase and red. (DOCX) [file pntd.0010965.s008.docx]

**S2 Table. *Clk* gRNA off-target sites in *Ae. albopictus* and *Cx. quinquefasciatus* genome**

| Species | Target Sequence | | Mismatches |
| --- | --- | --- | --- |
|  | **crRNA** | **DNA** |  |
| *Ae. albopictus* | ATGATAAGGATGACACCAAGNGG | AaGATAAGGATGACACCAttTGG | 3 |
|  | CAGATGCGTGAACTCCTCGTNGG | gAGATGCGTGAACTCtTCGTTGG | 2 |
| *Cx. quinquefasciatus* | CAAGAGTCACAACGAGATTGNGG | CAAGcGTCAgAACGAGATTcTGG | 3 |
|  | CGGCAAGTGCCCCAGCAGCGNGG | CGtggAGTGCCaCAGCAGCGTGG | 4 |

Note: Mismatches bases are highlighted in lowercase and red.
