# Supplementary material for: Clock genes regulate mating activity rhythms in the vector mosquitoes, Aedes albopictus and Culex quinquefasciatus
Source: PLoS Negl Trop Dis. 2022 Dec 1;16(12):e0010965. doi: 10.1371/journal.pntd.0010965 (PMC9746994; doi:10.1371/journal.pntd.0010965)
Supplement: S3 Table — (DOCX) [file pntd.0010965.s009.docx]

**S3 Table. Identity of cuticular hydrocarbon peaks of male adult *Ae. albopictus***

| Peak No. | Retention  time (min) | Hydrocarbon(s) |
| --- | --- | --- |
| 1 | 3.456 | nonane (C9) |
| 2 | 4.725 | n-decane (C10) |
| 3 | 5.672 | n-undecane(C11) |
| 4 | 6.494 | dodecane (C12) |
| 5 | 7.267 | n-tridecane (C13) |
| 6 | 7.983 | n-tetradecane (C14) |
| 7 | 8.661 | n-pentadecane (C15) |
| 8 | 9.376 | n-hexadecane (C16) |
| 9 | 10.166 | heptadecane (C17) |
| 10 | 11.051 | n-octadecane (C18) |
| 11 | 12.072 | n-nonadecane (C19) |
| 12 | 13.296 | eicosane (C20) |
| 13 | 14.458 | n-heneicosane (C21) |
| 14 | 15.711 | n-docosane (C22) |
| 15 | 17.071 | tricosane (C23) |
| 16 | 18.427 | n-tetracosane (C24) |
| 17 | 19.867 | n-pentacosane (C25) |
| 18 | 21.256 | hexacosane (C26) |
| 19 | 22.641 | n-heptacosane (C27) |
| 20 | 23.994 | octacosane (C28) |
| 21 | 25.321 | nonacosane (C29) |
| 22 | 26.628 | triacontane (C30) |
| 23 | 28.08 | n-hentriacontane (C31) |
| 24 | 29.776 | dotriacontane (C32) |
| 25 | 32.046 | tetratriacontane (C34) |
