# Supplementary material for: Clock genes regulate mating activity rhythms in the vector mosquitoes, Aedes albopictus and Culex quinquefasciatus
Source: PLoS Negl Trop Dis. 2022 Dec 1;16(12):e0010965. doi: 10.1371/journal.pntd.0010965 (PMC9746994; doi:10.1371/journal.pntd.0010965)
Supplement: S4 Table — (DOCX) [file pntd.0010965.s010.docx]

**S4 Table. Identity of cuticular hydrocarbon peaks of male adult *Cx. quinquefasciatus***

| Peak No. | Retention  time (min) | Hydrocarbon(s) |
| --- | --- | --- |
| 1 | 7.995 | n-tetradecane (C14) |
| 2 | 10.187 | heptadecane (C17) |
| 3 | 12.084 | n-nonadecane (C19) |
| 4 | 13.209 | eicosane (C20) |
| 5 | 14.441 | n-heneicosane (C21) |
| 6 | 15.744 | n-docosane (C22) |
| 7 | 17.104 | tricosane (C23) |
| 8 | 18.498 | n-tetracosane (C24) |
| 9 | 19.904 | n-pentacosane (C25) |
| 10 | 21.297 | hexacosane (C26) |
| 11 | 22.679 | n-heptacosane (C27) |
| 12 | 24.031 | octacosane (C28) |
| 13 | 25.358 | nonacosane (C29) |
| 14 | 26.686 | triacontane (C30) |
| 15 | 28.141 | n-hentriacontane (C31) |
| 16 | 29.804 | dotriacontane (C32) |
| 17 | 31.888 | tetratriacontane (C34) |
