# Supplementary material for: Clock genes regulate mating activity rhythms in the vector mosquitoes, Aedes albopictus and Culex quinquefasciatus
Source: PLoS Negl Trop Dis. 2022 Dec 1;16(12):e0010965. doi: 10.1371/journal.pntd.0010965 (PMC9746994; doi:10.1371/journal.pntd.0010965)
Supplement: S5 Table — (DOCX) [file pntd.0010965.s011.docx]

**S5 Table. The primers used in this study**

| Primer name | Sequences (5’-3’ ) | Annealing tem*per*ature and *cyc*les | Primer use |
| --- | --- | --- | --- |
| Aal *clk* F | ACGAGCTTCTATACAGTTTGGT | 55°C, 35 | Genotype identification for *Aalclk* with PCR |
| Aal *clk* R | TCGATCATACTCACTGAAGTACC |  |  |
| Cxq *clk* F | TCTCAGCGAGAAGAAACGGC | 55°C, 35 | Genotype identification for *Cxqclk* with PCR |
| Cxq *clk* R | AAACCATGTCGTAGACCGTCA |  |  |
| AalsgRNA37 OT-F | GGGATCATTTGGGAAGGGAGTT | 55°C, 35 | Off-target test for Aal sgRNA37 with PCR |
| AalsgRNA37 OT-R | CACTACGTCCAAGAGCCAACA |  |  |
| AalsgRNA248 OT-F | CACTTACAGGCAGATGACCCA | 55°C, 35 | Off-target test for Aal sgRNA248 with PCR |
| AalsgRNA248 OT-R | AAGAGCCACAACGAGATAGCG |  |  |
| CxqsgRNA170 OT-F | GACACACAGTTTCGCATTGGA | 55°C, 35 | Off-target test for Cxq sgRNA170 with PCR |
| CxqsgRNA170 OT-R | GACATAATACTGGGACGACGAG |  |  |
| CxqsgRNA323 OT-F | TTATCACGCAGGACGCTATCG | 55°C, 35 | Off-target test for Cxq sgRNA323 with PCR |
| CxqsgRNA323 OT-R | CGCAGCTCTAGTGTCCGTTC |  |  |
| Aal *clk* qF | GTCCCAATCCATCACCTGCA | 55°C, 40 | Expression profile analysis for *Aalclk* with qPCR |
| Aal *clk* qR | TGTTTGCGCTTGTTCTTCGG |  |  |
| Cxq *clk* qF | GAGTCACAACGAGATTGCGGT | 55°C, 40 | Expression profile analysis for *Cxqclk* with qPCR |
| Cxq *clk* qR | CCTCCAGGATCAGGTGGGTA |  |  |
| Aal *desat1* qF | TGCGACTACCAACCACAGTC | 55°C, 40 | Expression profile analysis for *Aaldesat1* with qPCR |
| Aal *desat1* qR | GCGGTAACTGCTGTAGTGGT |  |  |
| Cxq *desat1* qF | TAACACGTCTCCACCACAGC | 55°C, 40 | Expression profile analysis for *Cxqdesat1* with qPCR |
| Cxq *desat1* qR | TGGTGGTTTGGTCAGCTTCA |  |  |
| Aal rps7 qF | ATGAACTCGGACCTGAAG | 55°C, 40 | Reference gene for *Aal* control with qPCR |
| Aal rps7 qR | TTCTTGCTGTTGAACTCG |  |  |
| Cxq rps7 qF | TGGGACACGGAAAGCCATAC | 55°C, 40 | Reference gene for *Cxq* control with qPCR |
| Cxq rps7 qR | TTGATCAGCTGCGAACCGTC |  |  |
| Aal *desat1* T7F | GGATCCTAATACGACTCACTATAGGGACCGGTGTTCTGAACGAGA | 60°C, 35 | dsRNA synthesis of *Aaldesat1* |
| Aal *desat1* T7R | GGATCCTAATACGACTCACTATAGGCCAGGTGGCATTCAGGATGA |  |  |
| Cxq *desat1* T7F | GGATCCTAATACGACTCACTATAGGGCCGAAAAGCGCAAGATCAA | 60°C, 35 | dsRNA synthesis of *Cxqdesat1* |
| Cxq *desat1* T7R | GGATCCTAATACGACTCACTATAGGCAGGTTTCGCCCCAGAAGTA |  |  |
| GFP - T7F | GGATCCTAATACGACTCACTATAGGAATGGGCACAAATTTTCTGTCAGT | 55°C, 35 | dsRNA synthesis of GFP |
| GFP - T7R | GGATCCTAATACGACTCACTATAGGCCGGACTTGTATAGTTCATCCATGC |  |  |
