# Supplementary material for: Clock genes regulate mating activity rhythms in the vector mosquitoes, Aedes albopictus and Culex quinquefasciatus
Source: PLoS Negl Trop Dis. 2022 Dec 1;16(12):e0010965. doi: 10.1371/journal.pntd.0010965 (PMC9746994; doi:10.1371/journal.pntd.0010965)
Supplement: Attachment — Submitted filename: 20221011 Response to the reviewers.docx [file pntd.0010965.s012.docx]

**Response to the reviewers’ comments**

Key Review Criteria Required for Acceptance? As you describe the new analyses required for acceptance, please consider the following:

Methods

-Are the objectives of the study clearly articulated with a clear testable hypothesis stated? -Is the study design appropriate to address the stated objectives? -Is the population clearly described and appropriate for the hypothesis being tested? -Is the sample size sufficient to ensure adequate power to address the hypothesis being tested? -Were correct statistical analysis used to support conclusions? -Are there concerns about ethical or regulatory requirements being met?

Reviewer #1: I have concerns regarding the use of statistical tests that are not optimized for handling frequencies and percentages. Please find suggestions for alternative tests in my comments to the authors.

**Reply:** **Thanks for the suggestion and we have changed the statistical method (Line 276-281 of the revision). We assessed the mating rate data using a generalized linear model (GLM) with binomial distribution.**

Reviewer #2: Methods needs more details. Were the nighttime condition mosquitoes handled? Under dim red light? n # for experiments should be provided throughout.

For mating assays, how many groups were tested per experimental rep? Was power analysis performed?

**Reply:** **Thanks for the suggestion and we have added details to make the statement clearer and more precise. We handled the nighttime condition mosquitoes under dim red illumination (Line 144-145 of the revision). The numbers of mosquitoes for each experiment have been provided throughout.**

**In the mating rhythm experiment, we took 10 males and 10 females as a group. Eight independent groups were performed at eight zeitgeber times of the day. Each group was repeated three times. Each mosquito was measured only once, n=28-30 for each zeitgeber time (****Line 145 and line 349 of the revision). In the mating experiments with *desat1* RNAi, we took 30 males and 30 females as a group. Each treatment was replicated three times, n=88-90 for each treatment** **( Line 220-224 and 453-456 of the revision). For the mating experiments with CHC application, we took 20 males and 20 females as a group. Each treatment was replicated three times, n=59-60 for each treatment (Line 264-268 and 523-525 of the revision).**

**The sample sizes in the above mating assays referred to these studies (Wang G, Science. 2021; Jiwei Zhang, Communications biology, 2021; Takaomi Saka, PNAS, 2001). Yes, we performed power analysis by using IBM SPSS v. 27.0. Based on the sample size, we tested the power between 0.8 and 0.9 according to the preliminary experiments.**

How were the Clock mutants screened and confirmed? How were the target sites selected and were the mutant lines tested for off-target effects? Please provide details on how mutagenesis was determined and if the mosquitoes outcrossed before being tested.

**Reply: Thanks for the suggestion and we have provided relevant details in the revised manuscript (Line 166-192, S1 Fig, S2 Fig, S1 Table and S2 Table of the revision).**

**We designed primers upstream and downstream of the sgRNA target sites and screened the mosaic G_0_ by using mosquito genomic DNA isolated from leg and PCR. Mosaic G_0_ mosquitoes were mated with WT mosquitoes to obtain the G_1_ generation. Through PCR detection and sequence analysis, the G_1_ adults heterozygous with the same mutation type were identified and crossed with each other to produce the homozygous *clk* mutation strains (*Aalclk*^Δ293^ and *Cxqclk*^Δ98^). Homozygous *clk* mutants were confirmed with PCR.**

**We used CRISPOR (http://crispor.tefor.net), an online tool, to identify appropriate guide RNAs (sgRNAs) target sequence containing the T7 polymerase-binding site and specifically targeting 20 bp of the site. Exon 2 of *Ae. albopictus* and exon 3 of *Cx. quinquefasciatus* were used to design and determine optimum candidate sgRNAs. According to the core based off-target analysis, the top two, sgRNA1 and sgRNA2, were selected, which target *Aalclk* at positions 37 / 248 for *Ae. albopictus* and target *Cxqclk* at positions 170 / 323 for *Cx. quinquefasciatus*. Yes, all of the designed sgRNAs were checked using Cas-OFFinder online software to predict potential off-target sites. Off-target effects were tested in genomic DNA extracted from *clk* mutant strains. No off-target mutations were confirmed. Before being tested, all mosquito mutants were confirmed using PCR to be homozygous.**

Figure 3H&I and S2 B&D: More details of experimental conditions should be provided, including time (ZT) and duration of these experiments. How many females were examined?

**Reply: Thanks for the suggestion and we have added details of the experimental conditions. For the mating experiments with *desat1* RNAi, the mating assay was started at ZT9 and allowed to continue for 24 hours. We took 30 injected males and 30 virgin females as a group. A total of 88-90 female mosquitoes were dissected per treatment (Line 223-224, 453-456 of the revision). The same experiment was conducted between injected females and virgin males (Line 901-903 of the revision).**

What time of day was the CHC-applied mating assay performed for Ae. albopictus?

**Reply: We applied hydrocarbons to *Ae. albopictus* male at ZT6. For *Ae. albopictus*, three hours after perfuming, 20 treated male mosquitoes and 20 virgin female mosquitoes were exposed and allowed to mate for 2 hours.** **The mating time was from ZT9 to ZT11, which was the peak of mating activity (Line 264-268 and 523-525 of the revision).**

The authors nicely demonstrate here that there is a mating rhythm for both species tested. Authors should provide time of experiment for any mating assays performed.

**Reply:** **Thanks for the suggestion and we have added the details to make them clearer.**

**For the mating experiments with *desat1* RNAi, the mating assay was started at ZT9 and allowed to continue for 24 hours (Line 223-224 and 453-456 of the revision).**

**For the mating experiments with CHC application, for *Ae. albopictus,* the mating time was from ZT9 to ZT11. For *Cx. quinquefasciatus*, the mating assay was started at ZT9 and left overnight (Line 264-268 and 523-525 of the revision). Mating experiments were all performed during periods of peak mating activity. The different mating durations for *Ae. albopictus* and *Cx. quinquefasciatus* were used to try to achieve the mating rate at an intermediate level to help determine if CHCs promote or inhibit mosquito mating. The mating** **durations were decided after exploration in preliminary experiments.**

Variance in data is not very clear throughout this manuscript.

**Reply: We accepted the reviewer’s comment and made it clearer.** **In mating experiments, for error bars, we used 95% confidence intervals (CIs) instead of SEM.**

Reviewer #3: (No Response)

--------------------

Results

-Does the analysis presented match the analysis plan? -Are the results clearly and completely presented? -Are the figures (Tables, Images) of sufficient quality for clarity?

Reviewer #1: Yes.

Reviewer #2: Lines 93: Please elaborate on what the authors mean by ‘evolutionary mechanisms’ here.

**Reply: We removed the unclear description and reworded the introduction (Line 99-101 of the revision).**

When referring to time, Zeitgeber Time (ZT), rather than real time, should be provided.

**Reply:** **Thanks for the suggestion and we corrected this throughout the manuscript** **(Line 26 , Line 292, Line 314 of the revision, etc.).**

Line 239: 3 hours before darkness is ZT9, not ZT12. In Figure 1B, mating seems to peak at light:dark transition at ZT12. Either way, this sentence needs clarification.

**Reply:** **We corrected 3h before darkness to ZT9-12 (Line 295, 300 of the revision, etc.).**

Line 240: “~3h after lights off”. Is there reason for “~” here?  This should be referred to as ZT15.

**Reply: We changed the descriptions in the figures and results about zeitgeber time (such as ZT12-15) to describe the mating activity more precisely** **(Line 26, 314 of the revision, etc.).**

Line 243: Needs clarification. While the Ae. albopictus evening mating peak looks delayed by 3 hrs, the morning mating peak appears have advanced by 3 hrs and is less pronounced under DD condition compared to LD. Similarly, Culex seems to have a small morning mating peak at ZT0/24 under LD that is not observed under DD.

**Reply：Under LD conditions, there is a 12h:12h light/dark cycle to induce a 24h circadian rhythm. In the process of entrainment, the phase of the circadian clock, meaning its stage in the cycle relative to external time, is determined by environmental cues termed zeitgebers. When the** **zeitgeber is absent for a long time, the circadian clocks and the behaviors of organism may out of synchrony with the external environment.**

**Previously the mosquitoes used in the mating rhythm activity under DD condition were synchronized to** **DD condition for 4 days, 2 days after emergence. The lack of a 4-day entrainment in LD cycles and the absence of zeitgeber under DD for 4 days, may account for the difference in mating activity between DD and LD conditions. We supplemented the mating activity experiment on day 2 of DD after 4 days entrainment in LD, adapted from Takaomi Saka (PNAS, 2001). The results showed that the mating rhythm in the LDDD condition was similar to the trend in the LD condition of *Ae. albopictus* and *Cx. quinquefasciatus* (Line 142-144, 295-299, 313-314 and Fig 1 B&D of the revision).**

Lines 247-250: Is 4 days considered enough for re-entrainment to a complete anti-phasic condition (reversed schedule) before measuring mating frequency? The shift in timing of mating peak suggests that the animals may still be re-entraining.

**Reply: We chose to synchronize 4 days in mosquitoes based on the following literatures, Shetty V et.al. Sci Rep. (2022); Gustavo B. S. Rivas, (2018); Wang G, Science, (2021). According to the entrainment periods referenced therein, 4 days of entrainment under different light conditions should be sufficient for re-entrainment. Furthermore, our results showed that the mating rhythm in DLDD condition was similar to that after 4 days entrainment in DL, which is consistent with mating rhythm re-entrainment 4 days of entrainment in *Ae. albopictus* and *Cx. quinquefasciatus* (Line** **303-309, 316-317 and Fig 1 C&E of the revision).**

Line 248: Needs clarification. What do the authors mean by at each time period?

**Reply: We mean that the mating rate of *Ae. albopictus* was significantly higher at each time period during the day than that during the night.** **Because male and female mosquitoes are allowed to mate for three hours at each zeitgeber time. We changed the label on the abscissa and deleted the confusing sentence and reworded the description (Line 293-295 of the revision).**

Line 250-252: Lack of mating after lights on in Culex maybe due to acute suppression rather than re-entrainment. In order to demonstrate circadian re-entrainment of mating rhythm, the assay must be followed by sufficient re-entrainment period and then performed under free running condition (DD).

**Reply: We accepted the reviewer’s suggestion and supplemented the mating activity assay under constant darkness (DD) condition after reversed light cycle (DL) 4 days (Line 303-309, 316-317 and Fig 1 C&E of the revision).**

Line 265: While I agree with this conclusion, the way that DD data in Fig. 1E is presented does not seem to support this idea. How was day vs night total mating frequency calculated? Is ZT12 or ZT0 considered day or night?

**Reply: We reworked the mating activity experiment on day 2 of constant darkness (DD) after 4 days entrainment in LD or DL. The mating rhythms of mosquitoes under LDDD or DLDD were similar to those of mosquitoes under LD or DL cycles, respectively (Line 142-144, 295-299 and Fig 1 B-E of the revision). Furthermore, total mating rates during daytime (ZT0-12) and nighttime (ZT12-24) were calculated to evaluate the difference in mating activity between day and night.** **The results of the supplementary DD experiment support the conclusion that *Ae. albopictus* mainly mate during the day or subjective day and *Cx. quinquefasciatus* mainly mate at night under different conditions (Line 325-330 and Fig 1 F of the revision).**

**Under LD or LDDD condition, the total mating rate during daytime or subjective daytime (ZT0-12) was calculated as the number of inseminated females divided by the number of dissected females at zeitgeber times including ZT0-3, ZT3-6, ZT6-9 and ZT9-12. The total mating rate during nighttime (ZT12-24) was calculated as the number of inseminated females divided by the number of dissected females at zeitgeber times including ZT12-15, ZT15-18, ZT18-21 and ZT21-24. Under DL or DLDD conditions, the calculation of total mating rates for daytime and nighttime was the opposite of that for LD or LDDD (Line 152-158 and 322-325 of the revision). In the LD cycle, lights on at ZT 0 (8:00 am) and lights off at ZT12 (20:00 pm). In the DL cycle, lights on at ZT12 (20:00 pm) and lights off at ZT0 (8:00 am). In constant darkness (DD), lights off throughout the day (Line 138-141 of the revision).**

What are the authors’ interpretation on clock mRNA cycling in WT Culex? Here clock mRNA expression display 2-3 peaks, which is inconsistent with prior literature (Rivas 2018), and male versus female expression patterns look different from each other.

**Reply: The 2018 Rivas article showed that *clk* in *Ae. aegypti* had rhythmic oscillations. In their work, *Cx. quinquefasciatus* showed rhythmic disturbances under LDTC conditions and there were two zeitgebers, light/dark cycles and temperature fluctuations. In contrast, we used only light/dark cycles in our experimental conditions. These differences may account for the apparent discrepancies between the results in the two studies. The expression profiles of male and female mosquitoes were similar at other time points except ZT3. This difference may have been due to sample error. We therefore re-tested the samples from ZT3 and the ensuing expression profiles of male and female mosquitoes are now similar (Fig 2 G-H of the revision).**

Figure 2E & J: One reason for this could be because the animals are under LD condition, which can mask some of the effects circadian gene mutants can have. To measure circadian regulation, both mRNA abundance and mating frequency assays must be done under free running condition.

**Reply****:** **Thank you for the suggestion. We focused on experiments under LD conditions because our results have provided data to support the conclusion that the circadian rhythms of mating activity of *Ae. albopictus* and *Cx. quinquefasciatus* can be entrained to daily LD cycles. We measured the mating rhythms of mutant strains under LD condition and found that there was mating peak phase delayed to darkness in *Ae. albopictus* or mating peak phase inversion in *Cx. quinquefasciatus*. These results show that *clk* mutation can affect mating rhythm under the 12:12 LD cycles. However, whether there are other effects such as the direct effect by light exposure in mating rhythm, or whether other core clock genes have compensatory effects will be explored in further studies. We have added relevant comments in the discussion (Line 563-572 of the revision).**

How was the timepoint of 20:00 chosen for extraction of CHC? For figure 4C&D, how many days post dsRNA injection was the CHC collected? It’d be helpful to provide representative CHC profiles for dsRNA injected groups.

**Reply: ZT12 (20:00pm, light-dark transition) was selected for extraction of CHC because *Ae. albopictus* had a mating peak at** **ZT9-12, while *Cx. quinquefasciatus* also had mating activity at this time.** **We selected 4dpi with the best knockdown effect for CHC collection (Line 233, 468-471, and Fig 3 E-F of the revision). We supplemented representative CHC profiles for *desat1* dsRNA injected groups in the supplementary materials (S5 Fig).**

Reviewer #3: (No Response)

--------------------

Conclusions

-Are the conclusions supported by the data presented? -Are the limitations of analysis clearly described? -Do the authors discuss how these data can be helpful to advance our understanding of the topic under study? -Is public health relevance addressed?

Reviewer #1: Yes.

Reviewer #2: Discussion section summarizes main findings but lacks in scope. Authors should utilize this section to provide more insight.

**Reply: We followed the reviewer’s suggestion and added comments in the discussion (Line 565-578 and 594-604).**

Line 443-444: Needs clarification. Onset of daylight is ZT0/24. ZT3 is considered 3 hrs after daylight onset. Mating peak seems to be at ZT12, which at the transition of light/dark, not 3 hrs before.

**Reply:** **We corrected these descriptions to make them clearer.** **Here, we used ZT0-3 and ZT9-12 instead of the corresponding descriptions (Line 547 of the revision).**

Line 456-458: Needs clarification.

**Reply: These sentences were mainly intended to comment on the involvement of clock genes in behavioral activities. We removed the inappropriate reference and substituted “ The circadian clock is genetically controlled, and mutations in ‘clock genes’ can change rhythmic behavior in animals, including insects, humans and other species. The molecular clock is thought to control the expression of output genes throughout the body, thereby temporally controlling the behavior [5]”** **(Line 560-563 of the revision).**

Do the authors have any thoughts on the sex differences of desat knockdown effects on mating? Are the affected CHC more specific to males? Or could desat affect biosynthesis of CHC differently in each sex?

**Reply: We have added text to the discussion section to comment on this (Line 592-599 of the revision). “Mating is initiated by males in mosquitoes, and this is followed by mate recognition by the females during contact [44]. Recent work has shown that the desaturase gene *desat1* is up-regulated in swarming males and regulates the production of cuticular hydrocarbons [21]. Based on the role of males in mating and the results of our study, we propose that *desat1* plays an important role in CHCs synthesis in males and mating activity. However, whether there are other pheromone synthesis-related genes or receptors in female mosquitoes needs further research.”**

**We do not exclude the involvement of *desat1* in pheromone synthesis in female mosquitoes, but our results showed that *desat1* knockdown in females did not affect mating activity, suggesting that there may be other pheromone synthesis genes or receptors involved in pheromone and mating activity this sex. Another explanation may be possible sexual dimorphism of pheromones. Genes involved in pheromone synthesis or pheromone function may be different in each sex and this needs further study.**

Could the authors comment on what the implications of their findings may be in terms of vector control?

**Reply: We added text to the introduction and conclusion section. “** **Mosquito-control strategies, such as sterile insect technique (SIT) [23], that rely on manipulating or exploiting mating behaviors are rapidly becoming important tools in the management of mosquito populations. An in-depth understanding of the circadian characteristics of mating behavior and its mechanism in these two species may provide new insights into mosquito control.”（Line 101-105 of the revision). “The observed distinct mating activity rhythms of the two mosquito species and its molecular mechanisms regulated by clock genes and sex pheromones, may provide the basis for developing precise mosquito control strategies.” (Line 636-639 of the revision).**

Reviewer #3: (No Response)

--------------------

Editorial and Data Presentation Modifications? Use  this section for editorial suggestions as well as relatively minor modifications of existing data that would enhance clarity. If the only modifications needed are minor and/or editorial, you may wish to recommend “Minor Revision” or “Accept”.

Reviewer #1: NA

Reviewer #2: Use of red and green in the same graph is not recommended in order to keep graphs accessible to colorblind readers.

**Reply: We followed the reviewer’s suggestion and changed green to black (Fig 3 and S4 Fig of the revision).**

Incorrect figure and table references are found throughout. For example, reference to Figure S2 should be S1 in line 347 & 349; Figure S3 should be S2 in line 392; Table S3 should be Table S4 in line 176.

**Reply: We corrected this as the reviewer suggested (Line 206, 425, 427 and 479 of the revision).**

Line 147: “bodys” should be “bodies”

**Reply: Correct****ed (Line 195 of the revision).**

Line 213: I think the authors mean “3 hrs after perfuming”, rather than 3 hrs duration of perfuming.

**Reply: Yes and we corrected this (Line 264 of the revision).**

Formatting errors throughout: missing space after comma, between sentences, or before parenthesis.

**Reply: We edited to use appropriate space throughout the manuscript.**

Line 529: Perhaps the authors mean “that entrains to external light”?

**Reply: Yes and we corrected this (Line 635 of the revision).**

Reviewer #3: (No Response)

Summary and General Comments Use  this section to provide overall comments, discuss strengths/weaknesses of the study, novelty, significance, general execution and scholarship. You may also include additional comments for the author, including concerns about dual publication, research ethics, or publication ethics. If requesting major revision, please articulate the new experiments that are needed.

Reviewer #1: In the present manuscript, the authors analyzed the circadian nature of mating rhythms in two mosquito species of epidemiological importance: Aedes albopictus and Culex quinquefasciatus. Overall, the manuscript is well written and the logic flow between experiments is well articulated.

I have only 2 main concerns and a series of minor points and questions.

Main concerns:

Statistics: Instead of a chi-square (or Fisher) test, it would be more appropriate to use a test based on a binomial distribution. Ideally, a GLM with a binomial distribution of residuals or even better, a GLMM using the replicate number as a random effect.

**Reply: Thanks for the suggestion and we have changed the statistical method (Line 276-281 of the revision) and assessed the mating rate using a generalized linear model (GLM) with binomial distribution instead of a chi-square (or Fisher) test. Since each mosquito was tested only once and no repeated measurements were made on the same mosquito, we did not employ the GLMM model.**

Similarly, instead of t-tests to compare mating rates, a similar strategy based on a GLM or GLMM, with a binomial distribution, would be better.

**Reply: Thanks for the suggestion and we have changed the statistical method (Line 276-281 of the revision) and compared the mating rate between groups using a generalized linear model (GLM) with binomial distribution instead of t-tests.**

Finally, an ANOVA also assumes a normal distribution of the data and, by definition, frequencies and percentages are not normally distributed. I strongly recommend that authors use a GLM with binomial distribution of residuals and a posthoc Tukey test for pairwise comparisons between treatments.

**Reply: Thanks for the suggestion and we have changed the statistical method (Line 276-281 of the revision) and** **compared the mating rate at different zeitgeber times using a generalized linear model (GLM) with binomial distribution instead of ANOVA.** **Since there is no option for Turkey test in pairwise comparisons of generalized linear models in SPSS, we used the relatively conservative Sidak’s test for post hoc pairwise comparisons.**

The conclusions drawn from the reversed light cycle experiment are not clear. The authors state that these experiments demonstrate that the mating rhythm is entrainable, but since they were conducted under LD conditions, it could also simply be that a certain component of the rhythm is driven by external light conditions. An experiment where the light cycle is reversed for 3 days before testing mosquitoes under constant darkness conditions would be necessary to demonstrate that they have entrained to the reversed light cycle. Please clarify the interpretation of these results.

**Reply:** **We accepted the reviewer’s suggestion and supplemented the mating activity assay under constant darkness (DD) condition after reversed light cycle (DL) 4 days (Line 303-309, 316-317 and Fig. 1C&E of the revision).**

Minor points:

Abstract

L25: should “period” be plural? (since authors talk about the two transitions).

**Reply: Corrected (Line 26 of the revision).**

**.**L80: there is a missing “s” at D. simulans

**Reply: Corrected (Line 88 of the revision).**

**.**L89: in “Culex quinquefasciatus is a nocturnal, harassing mosquito”, what do you mean by harassing? Males are harassing females? Or that it is an aggressively anthropophilic mosquito?

**Reply: We accepted this comment and changed the word “harassing” with “anthropophilic.” (Line 94 of the revision).**

L89: replace “coordinately-guided” with “coordinated”

**Reply: We rewrote the description. “While a recent study showed that *An. gambiae* swarming and mating are coordinately-guided by clock genes, light, and temperature” has been changed with “ A recent study showed that knockdown of both *per* and *tim* in *An. gambiae* and *An. stephensi* caused a decrease in swarming and mating rate.” (Line 96-98 of the revision).**

L102: consider replacing “constructed” with “established”

**Reply: Corrected (Line 115 of the revision).**

L104: “ablation” should not be used here as this refers to RNAi. Instead, “the expression of the gene desat1 was inhibited” would be better.

**Reply: Corrected (Line 118-119 of the revision).**

L181: CHCs are extracted at 20:00pm. To what ZT time does this correspond?

**Reply: 20:00pm correspond to ZT12 under** **12:12 LD cycle (Line 233 of the revision).**

L196: replace “sec” with “a second”

**Reply: Corrected (Line 248 of the revision).**

L213: “After perfuming 3 hours,” did you mean “Three hours after perfuming,”?

**Reply: Yes and we corrected this (Line 264 of the revision).**

L239: shouldn’t ZT12 be ZT9? (i.e., 3 hours before lights off?)

**Reply: We corrected these descriptions and made the text clearer. Here, we used ZT0-3 and ZT9-12 instead of the corresponding descriptions (Line 295 of the revision).**

L251: “These results suggest that the circadian rhythms of mating activity can be precisely entrained by light signals.” Don’t you need to expose mosquitoes to this reversed light cycle and then transition to DD to show that they are entrained? Maybe what we are seeing is simply driven by external cues (light).

**Reply: We accepted the reviewer’s suggestion and supplemented the mating activity assay under constant darkness (DD) after reversed light cycle (DL) 4 days (Line 303-309, 316-317 and Fig. 1C&E of the revision).**

L263: “ There were no significant difference in the total mating rates of Ae. albopictus and Cx. quinquefasciatus between day and night under DD conditions (Fig 1E). These results indicate that the mating rhythms of the two species are completely opposite with Ae. albopictus mating during the day and Cx. quinquefasciatus mating at night.”

This paragraph is a bit unclear and needs to be reworded. What specific comparisons were made?

**Reply:** **We reworked the mating activity on day 2 of constant darkness after 4 days entrainment in LD or DL. The mating rhythms of mosquitoes under LDDD or DLDD were similar to those of mosquitoes under LD or DL cycles, respectively (Line 142-144, 295-299, 313-314 and Fig 1 B-E of the revision). According to the results of supplementary DD experiment, it supported the conclusion that *Ae. albopictus* mainly mated during the day or subjective day and *Cx. quinquefasciatus* mainly mated at night under different conditions (Line 325-330 and Fig 1 F of the revision).**

L310: “but there was no significant difference in daytime mating rates (P >0.05).” please add the values here for clarity.

**Reply: Thanks for the suggestion and we have added the values here for clarity (Line 378-379 of the revision).**

L444: again, in “onset of day-light (ZT3) and 3 hours before darkness (ZT12)” Shouldn’t it be ZT9?

**Reply: Thanks for the suggestion and we corrected to make it clearer. Here, we used ZT0-3 and ZT9-12 instead of the corresponding descriptions (Line 547 of the revision).**

L467: “tus” is missing from “quinquefascia”

**Reply: Corrected** **(Line 570 of the revision).**

L526: delete “in” from “that involve in the regulation”

**Reply: Corrected (Line 632 of the revision).**

Figure 1

Panels B-D: an ANOVA is used to compare frequencies while an ANOVA assume normal distribution. Using GLM with a binomial distribution and posthoc Tukey tests for pairwise comparisons would be better.

**Reply: We have changed the statistical method (Line 276-281 of the revision) and compared the mating rate at different zeitgeber times using a generalized linear model (GLM) with binomial distribution instead of ANOVA. Since there is no option for Turkey test in pairwise comparisons of generalized linear models in SPSS, we used the relatively conservative Sidak's test for post hoc pairwise comparisons.**

Panel D: So, with the reversed LD: what are we seeing? That rhythms are driven by light? Entrainment would require 3 days in reversed LD and then DD to see that they have entrained. The interpretation of these results needs to be clarified in the MS.

**Reply:** **We accepted the reviewer’s suggestion and supplemented the mating activity assay under constant darkness (DD) condition after reversed light cycle (DL) 4 days (Line 303-309, 316-317 and Fig 1C&E of the revision).**

E: Binomial tests would be more appropriate.

**Reply: We have changed the statistical method (Line 276-281 of the revision) and assessed the mating rate using a generalized linear model (GLM) with binomial distribution.**

Figure 2:

Panels D-E: So, although the difference between the scotophase of WT and clock knockouts seems to be different (but see my previous comment about statistics), the overall profile is similar between WT and mutants. Showing that mating rhythms are driven, to a large extent by light conditions. An interesting experiment would have been to test the knockout line under DD. Can authors comment on that in the manuscript? This would help readers identify the outstanding knowledge gaps.

Panels I-J:

**Reply: Thank you for the suggestion. We focused on experiments under LD conditions because our results have provided data to support the conclusion that the circadian rhythms of mating activity of *Ae. albopictus* and *Cx. quinquefasciatus* can be entrained to daily LD cycles. We measured the mating rhythms of mutant strains under LD condition and found that there was mating peak phase delayed to darkness in *Ae. albopictus* or mating peak phase inversion in *Cx. quinquefasciatus*. These results show that** ***clk* mutation can affect mating rhythm under the 12:12 LD cycles.** **However, whether there are other effects such as the direct effect by light exposure in mating rhythm, or whether other core clock genes have compensatory effects will be explored in further studies. We have added relevant comments to the discussion (Line 563-573 of the revision).**

Figure 3

Same comment here: additional DD experiments would have allowed the distinction between light and clock effects. Could authors add a comment in the discussion?

**Reply: Thank you for the suggestion. As above, *clk* mutation can affect mating rhythm and *desat1* genes expression under the 12:12 LD cycles. However, whether there are other effects such as the direct effect by light exposure in mating rhythm and downstream genes expression, or whether other core clock genes have compensatory effects as will be the subjects of further studies.** **The mechanisms of the interactions between *desat1*, clock genes and zeitgebers also need to be further explored.** **We have added relevant comments to the discussion (Line 600-604 of the revision).**

It is interesting that in males, the inversion of the curves in A and C leads to an increase in the mating rates but with a peak at the same times of day. Shouldn’t we expect to see a shift in the time of peak mating frequency? And a reduction of the mating rate during peak hours (since desat1 is less expressed)?

**Reply: When *desat1* was less expressed, the mating rates did not decrease at these time points. First, the *desat1* expression peak of WT did not coincide with the mating peak, and there was a phase time difference between them. For example, the expression of *desat1* peaked at ZT9 in WT *Cx. quinquefasciatus*, while the mating rate peaked at ZT12-15. Second, our next experiment proved that *desat1* was involved in the synthesis of CHCs, which in turn affected mating behavior. The *desat1* expression peak and mating peak did not coincide, the former occurred earlier than latter. One explanation is that the process is also involved in** **pheromone synthesis, mating activity initiation, mate recognition and other behaviors.** **Therefore, the peak or shift of *desat1* expression and mating activity may not occur at the same time. We have added comments to the discussion (Line 604-606 of the revision).**

Figure 4

One could argue that showing the unmated bars is redundant in panels F and G since the insemination rates already include that information. Please consider removing the grey bars.

**Reply:** **We removed the grey bars (Fig 4 F-G of the revision).**

Reviewer #2: Liu et al. investigate an interesting topic of circadian regulation of mating activity in Aedes albopictus and Culex quinquefasciatus mosquitoes. This reviewer agrees with the general conclusion and find the study interesting. There are parts that require clarification and discussion section lacks insight in its current state. Few of the conclusions are not supported by their data and need to be clarified.

**Reply: We added this description in discussion (Line 565-578, 594-604 of the revision ) and conclusion (Line 634-637 of the revision ).**

**We repeated the experiment with the contradictory results. Specifically, we repeated the mating activity on day 2 of constant darkness after 4 days entrainment in LD or DL. The mating rhythms of mosquitoes under LDDD or DLDD were similar to those of mosquitoes under LD or DL cycles, respectively (Line 295-299, 313-314 and Fig 1 B-E of the revision). The results of the supplementary DD experiment supported the conclusion that *Ae. albopictus* mainly mated during the day or subjective day and *Cx. quinquefasciatus* mainly mated at night under different conditions (Line 325-330 and Fig 1 F of the revision).**

Reviewer #3: The authors have done a great job of presenting the regulation of circadian mating activity in Aedes albopictus and Culex quinquefasciatus by clock genes. This study is quite important as it adds to the current body of knowledge of mating behavior and circadian rhythm of mosquitoes, and also provide insights for the development of mosquito control programs. The paper was clearly written, and the methods were well described. I have no big concerns with this manuscript; however, I would like to provide some few suggestions that will strengthen the manuscript.

1. This current study is similar to a previous investigation by Wang et al., 2021 (Clock genes and environmental cues coordinate Anopheles pheromone synthesis, swarming, and mating). In the 2021 study, the role of the clock genes (period and timeless) and desat1 was evaluated in three Anopheles species. While a different clock gene was investigated in two different mosquito species in this study, the authors need to state clearly a significant research gap this study is trying to address in mosquito mating behavior. I do not think the justification provided in the introduction was strong enough.

**Reply: We added text to the description****s in introduction (Line 97-105 of the revision).**

2. Line 90-91, “…the mating activity of Ae. albopictus and Cx. quinquefasciatus has yet to be determined molecula”l–" - is the use of "has" correct in this context?

**Reply:** **We reworded these sentences to make the statement more precise. “** **However, it remains unclear how the circadian clock regulates the mating behavior of *Ae. albopictus* and *Cx. quinquefasciatus*, two mosquito species with different activity patterns. ”** **(Line 99-101 of the revision).**

3. There are some formatting issues in Lines 110, 114, 128 etc. The authors might want to do a thorough check through the whole manuscript.

**Reply: We have** **checked thoroughly the whole manuscript for such errors.**

4. Line 147 - "bodys" should be "bodies".

**Reply****: Corrected (Line 195 of the revision).**

5. Lines 179 and 184 – The unit of measurement should be confirmed and corrected accordingly.

**Reply: Corrected** **(Line 232-235 of the revision).**

6. Line 210-211, "Every mosquito was applied ~1μL." - This is not clear.

**Reply: We rewrote the sentence to make it clearer. “****Hydrocarbons were dissolved in n-hexane at a concentration of 75 µg/mL [21] and paintbrushes were used to apply 1****μL to the abdomen of two-day-old male *Ae. albopictus* and *Cx. quinquefasciatus* mosquitoes.” (Line 260-262 of the revision).**

7. Line 215-217 - Is there a reason for the difference in the mating duration for the two mosquito species? If yes, sufficient justification should be provided.

**Reply: Yes, and we added the reasoning to the methods. The different mating durations for *Ae. albopictus* and *Cx. quinquefasciatus* were used to try to achieve the mating rate at an intermediate level to help determine if CHCs promote or inhibit mosquito mating. The mating durations were decided after exploration in preliminary experiments (Line 269-271 of the revision).**

8. Line 275-276 (Figure 1 legend): No description of the gray portion of the bar.

**Reply: We have added to the description. Gray portion indicates “subjective day” under DD condition (Line 345 of the revision).**

9. Line 303 – “Circadian” should be starting with a lowercase “c”.

**Reply: Corrected (Line 372 of the revision).**

10. Line 391 - compared should replace "comparted".

**Reply: Corrected (Line 479 of the revision).**

--------------------

PLOS authors have the option to publish the peer review history of their article (<a href="<https://urldefense.com/v3/__https://journals.plos.org/plosntds/s/editorial-and-peer-review-process*loc-peer-review-history__>;Iw!!CzAuKJ42GuquVTTmVmPViYEvSg!P25ZtZpZiwuEnScy-Ky6k2lUH0h2Hp-ZblV70AD--6oA59j0Klvn_nIxB8i_ZiV12tsjxVh01o07u8Y$  " target="_blank">what does this mean?</a>). If published, this will include your full peer review and any attached files.

If you choose “no”, your identity will remain anonymous but your review may still be made public.

Do you want your identity to be public for this peer review? For information about this choice, including consent withdrawal, please see our <a href="<https://urldefense.com/v3/__https://www.plos.org/privacy-policy__>;!!CzAuKJ42GuquVTTmVmPViYEvSg!P25ZtZpZiwuEnScy-Ky6k2lUH0h2Hp-ZblV70AD--6oA59j0Klvn_nIxB8i_ZiV12tsjxVh0H2CESlk$  " target="_blank">Privacy Policy</a>.

Reviewer #1: No

Reviewer #2: No

Reviewer #3: No
