# Supplementary material for: Clock genes regulate mating activity rhythms in the vector mosquitoes, Aedes albopictus and Culex quinquefasciatus
Source: PLoS Negl Trop Dis. 2022 Dec 1;16(12):e0010965. doi: 10.1371/journal.pntd.0010965 (PMC9746994; doi:10.1371/journal.pntd.0010965)
Supplement: Attachment — Submitted filename: 20221117 Response to the reviewers.docx [file pntd.0010965.s013.docx]

**Response to the reviewers’ comments**

The reviewer have indicated that the paper is improved, but there are still minor issues that need to be addressed.

Reviewer's Responses to Questions

<b>Key Review Criteria Required for Acceptance?</b></br></br>
As you describe the new analyses required for acceptance, please consider the following:</br></br>

<b>Methods</b></br></br>
-Are the objectives of the study clearly articulated with a clear testable hypothesis stated?</br>
-Is the study design appropriate to address the stated objectives?</br>
-Is the population clearly described and appropriate for the hypothesis being tested?</br>
-Is the sample size sufficient to ensure adequate power to address the hypothesis being tested?</br>
-Were correct statistical analysis used to support conclusions?</br>
-Are there concerns about ethical or regulatory requirements being met?</br></br>

Reviewer #1: See below

Reviewer #2: (No Response)

Reviewer #3: The methods used in this study are adequate for the hypothesis being tested and suitable for the study system.

--------------------

<b>Results</b></br></br>
-Does the analysis presented match the analysis plan?</br>
-Are the results clearly and completely presented?</br>
-Are the figures (Tables, Images) of sufficient quality for clarity?</br></br>

Reviewer #1: See below

Reviewer #2: (No Response)

Reviewer #3: Results were properly presented and described.

--------------------

<b>Conclusions</b></br></br>
-Are the conclusions supported by the data presented?</br>
-Are the limitations of analysis clearly described?</br>
-Do the authors discuss how these data can be helpful to advance our understanding of the topic under study?</br>
-Is public health relevance addressed?</br></br>

Reviewer #1: See below

Reviewer #2: (No Response)

Reviewer #3: Important conclusions and inferences made in this study are consistent with the data presented. Public health importance of this study was properly addressed.

--------------------

<b>Editorial and Data Presentation Modifications?</b></br><br/>
Use  this section for editorial suggestions as well as relatively minor modifications of existing data that would enhance clarity. If the only modifications needed are minor and/or editorial, you may wish to recommend “Minor Revision” or “Accept”.

Reviewer #1: none

Reviewer #2: (No Response)

Reviewer #3: The authors might need to modify the sentence on lines 262-263 to read as follows: "Mosquitoes which received no solvent (blank group) and those that only got the n-hexane application (positive control) were included for comparisons".
**Reply: Thanks for the suggestion and we have modified the corresponding sentence according to your suggestion (Line 275-276 of the revision).**
--------------------

<b>Summary and General Comments</b></br></br>
Use  this section to provide overall comments, discuss strengths/weaknesses of the study, novelty, significance, general execution and scholarship. You may also include additional comments for the author, including concerns about dual publication, research ethics, or publication ethics. If requesting major revision, please articulate the new experiments that are needed.

Reviewer #1: Overall, the authors have addressed the comments of the reviewers and added experimental details to the manuscript. Concerns about the statistical approach employed in the study have been addressed by using GLMs based on binomial distributions.

However, in the first part of the results section, the authors use wording suggesting that the GLM proves “distinct rhythmicity“. Rhythmicity needs to be tested by other statistical means. That being said, the authors could change the wording to “....activities were significantly influenced by the time of day”. Or something to this effect.
**Reply: Thanks for the suggestion and we have changed the corresponding sentence according to your suggestion (Line 307-308, 324-325 of the revision)**

L291: if measured under LD conditions, these are not “circadian” mating activities, but diel rhythms since they result from a combination of endogenous control and exogenous drive.
**Reply: Thanks for the suggestion and we accepted the reviewer’s comment. We have changed the description about “ circadian ” to “ diel ” under LD conditions (Line 305 of the revision).**

Reviewer #2: I thank the reviewers for their effort in addressing questions and concerns in the initial review. Two major concerns remain: (1) Results presented in Figures 2 and 3 still cannot conclude circadian effects as they were performed under LD. This is consistent with Reviewer #1’s initial concern. (2) Clk mutant mosquitoes were not properly outcrossed. Doing so is standard and necessary to rule out any genetic background effects, especially for behavioral tests. Both of these points should be clearly and properly addressed in the final version of this manuscript.
**Reply:**

**(1) Thanks for the suggestion and we accepted the reviewer’s comment. As Reviewer #1’s suggestion, if measured under LD conditions, these are not “circadian” mating activities, but diel rhythms resulted from a combination of endogenous control and exogenous drive. It should be diel rhythm in the presence of a light-dark (LD) cycle. So we used “diel” instead of “circadian” to make the descriptions more precise and we emphasize the influence of *clock* gene on temporal expression of *desat1* gene and diel mating behavior rhythm under LD cycles. We have** **modified the conclusion as “Their diel mating rhythms are regulated by their respective orthologs of the core clock gene *clk* and the pheromone synthesis-related *desat1* gene under LD cycles” (Line 543-549, 642-645 of the revision)*.***

**(2) The methods of establishing *clk* mutant strain that we described earlier was too simple and inappropriate. Therefore, we described the outcrossed method in detail (Line 177-197 of the revision). The larvae hatched from all G_0_ generation embryos after microinjection were reared to adult mosquitoes, and the male and female had been separated in the pupal stage.** **Genomic DNA was extracted from legs of individual adults and** **mosaic G_0_ mosquitoes were screened by PCR.** **Subsequently, each mosaic G_0_ virgin male was mated with three wild-type virgin females. Meanwhile, each mosaic G_0_ virgin female was mated with three wild-type virgin males.** **Female mosquitoes were fed blood to lay eggs and G_1_ generation was obtained.** **The genomic DNA was extracted from legs of individual G_1_ adult mosquitoes and the heterozygous G_1_ adults were detected by** **PCR. The PCR products were purified and analyzed by sequencing.** **Through sequence analysis, the G_1_ adults heterozygous male and female mosquitoes with the same mutation type were screened and crossed with each other to produce G_2_ generation.** **The homozygous *clk* mutants were selected from G_2_ generation by the same detection and sequence analysis. These homozygous *clk* mutants were mass-crossed to establish the *clk* mutant strain.**

Overall, this manuscript has been largely improved with interesting and important findings. With the two major points listed above addressed properly, I would recommend this manuscript for publication.

Reviewer #3: I appreciate the authors for the clarifications provided to the concerns raised and the clear responses to the reviewers' comments. The study is important as it provides evidence to the role of the circadian clock in the mating behavior of Aedes albopictus and Culex quinquefasciatus. Conclusions derived from this study are relevant for devising novel mosquito control strategies. This study will consequently expand knowledge in the field of circadian biology and integrated pest management which is important not only to scientists in these fields, but also the general public. Therefore, I recommend it for publication.

--------------------

PLOS authors have the option to publish the peer review history of their article (<a href="<https://journals.plos.org/plosntds/s/editorial-and-peer-review-process#loc-peer-review-history>" target="_blank">what does this mean?</a>). If published, this will include your full peer review and any attached files.<br><br>

If you choose “no”, your identity will remain anonymous but your review may still be made public.<br><br>

<b>Do you want your identity to be public for this peer review?</b> For information about this choice, including consent withdrawal, please see our <a href="<https://www.plos.org/privacy-policy>" target="_blank">Privacy Policy</a>.

Reviewer #1: No

Reviewer #2: No

Reviewer #3: Yes: Oluwaseun M. Ajayi


Figure Files:
While revising your submission, please upload your figure files to the Preflight Analysis and Conversion Engine (PACE) digital diagnostic tool, [https://pacev2.apexcovantage.com.](https://pacev2.apexcovantage.com./) PACE helps ensure that figures meet PLOS requirements. To use PACE, you must first register as a user. Then, login and navigate to the UPLOAD tab, where you will find detailed instructions on how to use the tool. If you encounter any issues or have any questions when using PACE, please email us at [figures@plos.org](mailto:figures@plos.org).
 **Reply: Done.**

Data Requirements:
Please note that, as a condition of publication, PLOS' data policy requires that you make available all data used to draw the conclusions outlined in your manuscript. Data must be deposited in an appropriate repository, included within the body of the manuscript, or uploaded as supporting information. This includes all numerical values that were used to generate graphs, histograms etc.. For an example see here: <http://www.plosbiology.org/article/info%3Adoi%2F10.1371%2Fjournal.pbio.1001908#s5.>
 **Reply: The authors confirm that all data underlying the findings are fully available without restriction. All relevant data are within the paper and its Supporting Information files.**

Reproducibility:
To enhance the reproducibility of your results, we recommend that you deposit your laboratory protocols in protocols.io, where a protocol can be assigned its own identifier (DOI) such that it can be cited independently in the future. Additionally, PLOS ONE offers an option to publish peer-reviewed clinical study protocols. Read more information on sharing protocols at <https://plos.org/protocols?utm_medium=editorial-email&utm_source=authorletters&utm_campaign=protocols>

References
Please review your reference list to ensure that it is complete and correct. If you have cited papers that have been retracted, please include the rationale for doing so in the manuscript text, or remove these references and replace them with relevant current references. Any changes to the reference list should be mentioned in the rebuttal letter that accompanies your revised manuscript. If you need to cite a retracted article, indicate the article's retracted status in the References list and also include a citation and full reference for the retraction notice.
 **Reply: Done.**

__________________________________________________
In compliance with data protection regulations, you may request that we remove your personal registration details at any time.  (Use the following URL: <https://www.editorialmanager.com/pntd/login.asp?a=r>). Please contact the publication office if you have any questions.
